# Solitary Fibrous Tumor of the Shoulder Metastasizing to the Small Intestine Presenting as Intussusception: A Case Report

**DOI:** 10.7759/cureus.42235

**Published:** 2023-07-21

**Authors:** Hasan Arafat, Amer Zughayyar

**Affiliations:** 1 Cancer Care Center, Augusta Victoria Hospital, Jerusalem, PSE

**Keywords:** solitary fibrous tumor, malignancy, rare vascular tumor, rare tumors, ileo-ileal intussusception, solitary fibrous tumor (sft)

## Abstract

Solitary fibrous tumors (SFTs) are rare vascular malignancies that are rarely reported in the upper limb, and even rarer as secondary metastasis to the small bowel. We present a case of a 39-year-old male patient, a known case of metastatic SFT, presenting with severe abdominal pain and vomiting. Computed tomography showed ileo-ileal intussusception. Subsequently, he underwent surgical resection. Histopathological examination showed high-grade sarcoma of the intestinal wall, confirming small intestinal metastasis. This constitutes one of the rare cases of SFTs metastasizing to the small bowel, specifically to the ileum. A review of the literature on similar cases is presented. There appears to be a connection between upper limb SFT and bowel metastasis according to reported literature. However, the scarcity of similar reports makes the generalizability of the conclusion limited.

## Introduction

Solitary fibrous tumors (SFTs) are rare vascular malignancies. They derive from Zimmerman’s pericytes, a type of modified myocytes that line capillaries, postcapillary venules, and sinusoidal spaces [1]. The pleura is the most common site. Extrathoracic SFTs are very rare, and those involving the upper limbs are even rarer [2]. The natural history is typically indolent and the prognosis is good. Primary sites of metastasis are the pleura, meninges, and extrathoracic soft tissues. Various other metastatic sites have been documented in the existing literature [3].

Here, we report a rare case of a 39-year-old male patient with right shoulder SFT with metastasis to the small bowel, presenting as intussusception.

## Case presentation

A 39-year-old male patient from Palestine, with a free past medical and past surgical history, presented to the orthopedic clinic in August 2018 with a right shoulder mass that started to increase in size six months prior, with no associated limitation of movement or pain. The mass was resected but was not sent for pathology. Four months later, the mass recurred, so it was resected again and sent for histopathological examination. Results showed a malignant SFT, with a mitotic factor higher than 5/10 by high-pass filter (HPF) and atypical mitosis. Magnetic resonance imaging (MRI) showed an area of 6 cm residual mass invading the superficial fascia. Chest-abdomen-pelvis computed tomography (CT) showed no distant metastasis.

Forty-five greys (Gy) (1.8 Gy daily dose for 25 fractions) of neoadjuvant radiation were given to the patient prior to surgical excision of the residual tissue. This was followed by four cycles of adjuvant IA protocol (ifosfamide (2500 mg/m^2^), mesna, (800 mg/m^2^), and doxorubicin (25 mg/m^2^) intravenously (IV) for three days every three weeks). The first assessment showed no evidence of disease (NED), so the patient was kept on follow-up.

**Figure 1:**
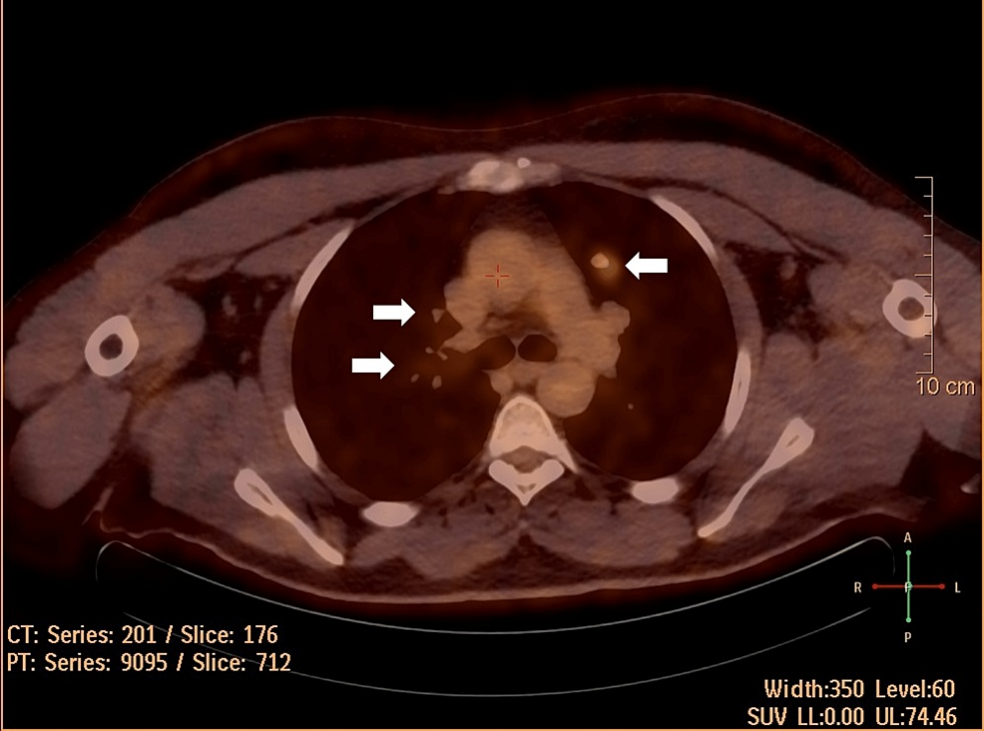
Axial view of PET/CT done on May 2020. White arrows point to multiple lung nodules representing metastasis. PET/CT: positron emission tomography/computed tomography.

Positron emission tomography/computed tomography (PET/CT) in May 2020 showed left lung hypermetabolic nodules suggestive of lung metastasis, as shown in Figure 1. The patient was sent for thoracic surgery. Histopathologic examination of excised nodules showed SFT. A new PET/CT in September 2020 showed NED. The patient was kept on regular follow-ups.

PET/CT in March 2021 showed new right lung nodules with lytic deposits in the fourth right rib and left pubic bone, suggesting recurrence. The case was discussed in the weekly multidisciplinary tumor board, and it was agreed that the patient should undergo lung metastasectomy, followed by two cycles of IE protocol, stereotactic body radiation (STBR) to the left pubic bone, and then four more cycles of IE. The patient received the first cycle of IE (ifosfamide 1800 mg/m2 with etoposide 100 mg/m2 once daily in addition to mesna 600 mg/m2 three times per day IV for five days), which was complicated by generalized weakness and fatigue, so the dose was reduced by 20%. The second cycle was complicated by anemia, necessitating admission for blood transfusion. The patient was then evaluated in the radiation oncology clinic, and simulation showed that the mass had increased in size, and orthopedic surgery disfavored surgical intervention, so four more cycles of IE were administered in addition to zoledronic acid, 4 mg IV monthly, due to bone metastasis. Twenty Gy in five fractions was administered as a means of palliative radiation. Whole body CT in December 2021 showed disease progression (DP), so the regimen was switched to pazopanib, 800 mg orally once daily. After three months of pazopanib therapy, he presented on March 2022 to the hospital complaining of increasing pain in the right hip and back. The patient was admitted for pain control and underwent his second assessment while admitted. Imaging was suggestive of DP with new metastatic lesions to the sixth thoracic vertebra and mild spinal cord compression. Pazopanib was stopped, and 20 Gy in four fractions of palliative radiotherapy to the spine and right rib were administered. The line was switched to temozolomide (150 mg/m2 orally on days one to seven and 15 to 21) and bevacizumab (5 mg/kg IV on days eight and 22) monthly.

**Figure 2:**
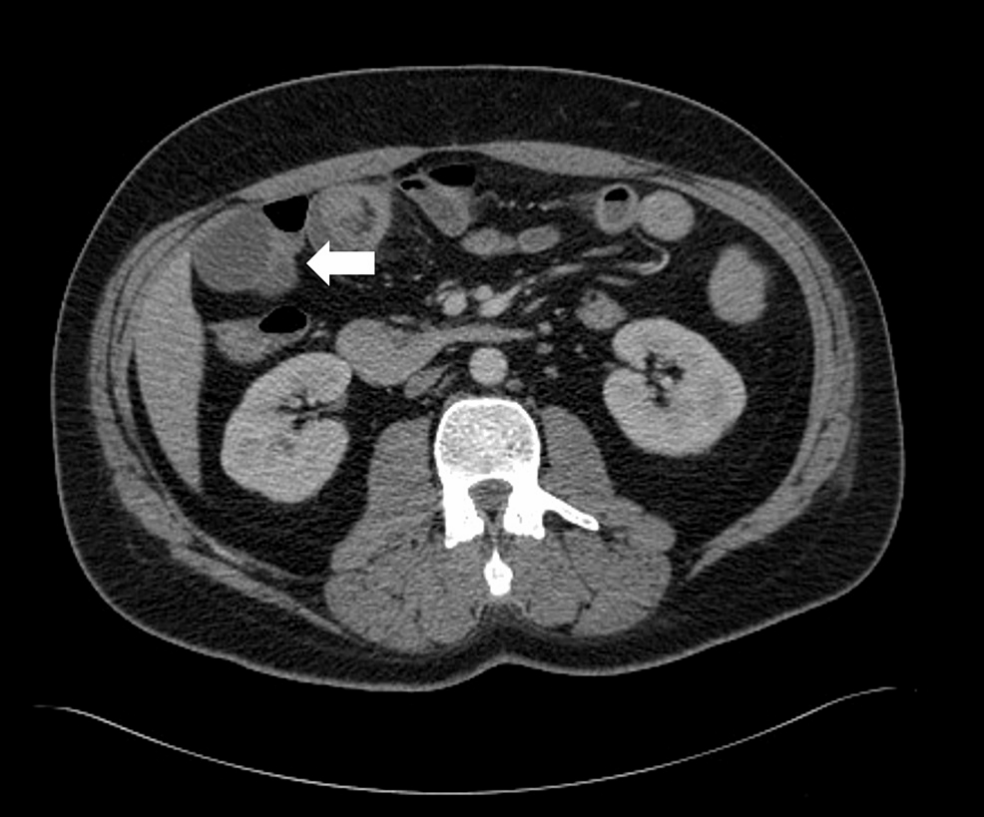
Axial contrast-enhanced CT image obtained at presentation with abdominal pain, showing evidence of target-like appearance in the subhepatic short segment of the small bowel measuring about 2.7 cm indicating small bowel intussusception.

**Figure 3:**
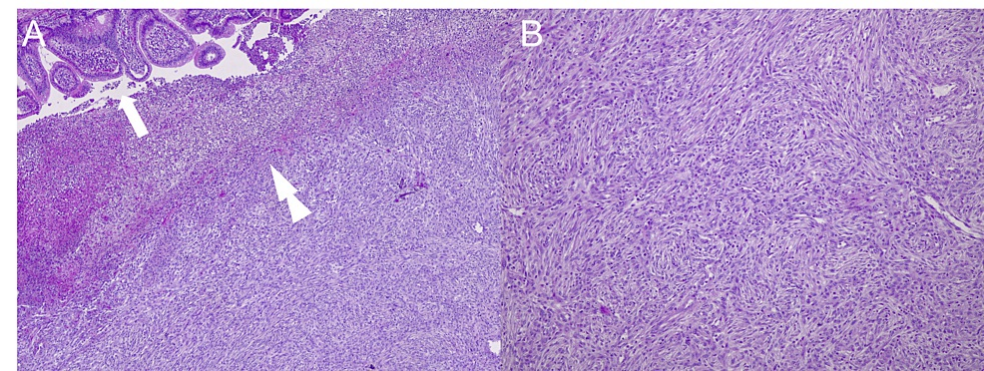
H&E stain of the malignant solitary fibrous tumor excised from the site of ileo-ileal intussusception. (A) 10x magnification of the excised tumor, which demonstrates the involvement of the intestinal wall with the tumor. There is ulceration from tumor invasion, pointed at by the double arrow. The white arrow points at the normal intestinal lumen. (B) H&E stain 20x magnification shows spindle cells with moderate-to-severe nuclear atypia and brisk mitotic figures. H&E: hematoxylin and eosin.

Prior to receiving the first cycle, the patient mentioned new-onset mild upper gastrointestinal bleeding, so bevacizumab was omitted for one cycle and the patient was scheduled for upper endoscopy. The patient returned to the hospital due to recurrent vomiting and severe epigastric pain that was progressive in nature. The patient was hypotensive (92/55 mmHg), tachycardic (110 beats per minute), and afebrile, but looked ill and lethargic. Abdominal examination disclosed a tender abdomen with no rigidity. Routine labs showed a drop in hemoglobin (8 mg/dL). An abdominopelvic CT scan (Figure 2) showed ileo-ileal intussusception. The patient underwent laparotomy, with resection of the terminal ileum and ileo-ileal anastomosis. The resected segment was sent for pathology, showing high-grade sarcoma of the intestinal wall, confirming small intestinal metastasis. Histopathology is shown in Figure 3. The patient's condition stabilized following the procedure. He was discharged in good condition and continued on the same line.

## Discussion

SFTs are a type of mesenchymal tumor. They affect both sexes equally and classically present in the fifth to sixth decades [4]. Despite their classical intrathoracic location, they are now known to occur as frequently in extra-thoracic locations [5]. The anatomic site of the primary tumor dictates its behavior, with those occurring in limbs behaving more aggressively compared to other sites. This is surprising due to the fact that such localizations are easier to operate on, giving better local control, which is assumed to limit the metastatic process [6]. In a case series studying extrathoracic, extrameningeal SFT, it was found that tumors of the upper limb made only 16.7% of the studied cases, with those of the lower limb making 41.7%, the torso making 8.3%, and the pelvis making 33.3% [5]. Histopathological features suggestive of malignant behavior include increased cellularity, nuclear pleomorphism, high HPF, and cellular atypia [7]. SFT of the upper extremity is rare. In 2012, the authors reported a case in the dorsal region of the hand, and another case of SFT of the shoulder was reported in 2022, with only a handful of similar cases existing in the literature [8]. Metastasis to the gastrointestinal tract is rare. A case of central nervous system (CNS) SFT metastatic to the pancreas was recorded in 2022, being asymptomatic and found incidentally during imaging. Rare cases of SFT have been reported in the ileum but as primary tumors [9]. Secondary SFT of the bowel is very rare. A single case of SFT metastatic to the appendix was recorded in 2017, with the primary lesion originating from the musculoskeletal tract [10]. The issue of whether intestinal metastasis tends to be of musculoskeletal origin needs to be explored more.

## Conclusions

In summary, we came across a rare case of shoulder SFT, with an even rarer site of metastasis and presentation (metastasis to the ileum presenting with intussusception). We have to know that SFT can metastasize to the small bowel and present with intussusception. Moreover, there seems to be an association between upper limb tumors and metastasis to the small bowel. More experience is needed to understand the biological behavior of SFT. Metastatic SFT should be included in the differential diagnosis of intussusception.
